# Cost and impact of policies to remove and reduce fees for obstetric care in Benin, Burkina Faso, Mali and Morocco

**DOI:** 10.1186/s12939-016-0412-y

**Published:** 2016-08-02

**Authors:** S. Witter, C. Boukhalfa, J. A. Cresswell, Z. Daou, V. Filippi, R. Ganaba, S. Goufodji, I. L. Lange, B. Marchal, F. Richard, On behalf of the FEMHealth team

**Affiliations:** 1 Immpact programme, University of Aberdeen, Aberdeen, AB25 2ZD Scotland, UK; 2 ENSP, Rue Lamfadel Cherkaoui, Madinat Al Irfane, BP: 6329, Rabat, Morocco; 3 MARCH Centre for Maternal, Adolescent, Reproductive & Child Health, London School of Hygiene & Tropical Medicine, London, WC1E 7HT UK; 4 MARIKANI, BP 2753, Rue 600, Porte 335 Baco djicoroni, ACI Bamako, Mali; 5 Department of Infectious Diseases Epidemiology, London School of Hygiene and Tropical Medicine, Keppel Street, WC1E 7HT London, UK; 6 AFRICSanté, 773 Rue Guillaume Ouédraogo, BP 298, Bobo-Dioulasso, Burkina Faso; 7 Centre de Recherche en Reproduction Humaine et en Démographie, 06BP567 Cotonou, Benin; 8 Health Services Organisation unit, Department of Public Health, Institute of Tropical Medicine, Nationalestraat 155, 2000 Antwerp, Belgium; 9 Unit of Maternal and Reproductive Health, Public Health Department, Institute of Tropical Medicine, Nationalestraat 155, 2000 Antwerp, Belgium; 10 ReBUILD, Institute for Global Health and Development, Queen Margaret University, Edinburgh, EH21 6UU Scotland, UK

**Keywords:** Exemptions, User fees, Deliveries, Caesareans, Maternal health, Benin, Burkina Faso, Mali, Morocco

## Abstract

**Background:**

Across the Africa region and beyond, the last decade has seen many countries introducing policies aimed at reducing financial barriers to obstetric care. This article provides evidence of the cost and effects of national policies focussed on improving financial access to caesarean and facility deliveries in Benin, Burkina Faso, Mali and Morocco.

**Methods:**

The study uses a comparative case study design with mixed methods, including realist evaluation components. This article presents results across 14 different data collection tools, used in 4–6 research sites in each of the four study countries over 2011-13. The methods included: document review; interviews with key informants; analysis of secondary data; structured extraction from medical files; cross-sectional surveys of patients and staff; interviews with patients and observation of care processes.

**Results:**

The article finds that the policies have contributed to continued increases in skilled birth attendance and caesarean sections and a narrowing of inequalities in all four countries, but these trends were already occurring so a shift cannot be attributed solely to the policies. It finds a significant reduction in financial burdens on households after the policy, suggesting that the financial protection objectives may have been met, at least in the short term, although none achieved total exemption of targeted costs. Policies are domestically financed and are potentially sustainable and efficient, and were relatively thoroughly implemented. Further, we find no evidence of negative effects on technical quality of care, or of unintended negative effects on untargeted services.

**Conclusions:**

We conclude that the policies were effective in meeting financial protection goals and probably health and equity goals, at sustainable cost, but that a range of measures could increase their effectiveness and equity. These include broadening the exempted package (especially for those countries which focused on caesarean sections alone), better calibrated payments, clearer information on policies, better stewardship of the local health system to deal with underlying systemic weaknesses, more robust implementation of exemptions for indigents, and paying more attention to quality of care, especially for newborns.

## Résumé

**Introduction:**

Ces dix dernières années de nombreux pays africains ont introduit des politiques visant à réduire les barrières financières aux soins obstétricaux. Cet article fournit des informations sur les coûts et les effets des politiques nationales axées sur l’amélioration de l’accès financier aux césariennes et plus largement aux accouchements au Bénin, Burkina Faso, Mali et Maroc.

**Méthodes:**

Une étude comparative d’études de cas, avec des composantes d’évaluation réaliste, a été réalisée en utilisant des méthodes mixtes (quantitatives et qualitatives). Cet article présente les résultats de 14 outils de collecte différents, utilisés dans 4 à 6 sites de recherche dans chacun des quatre pays de l’étude entre 2011 et 2013.

**Résultats:**

L’étude a montré une augmentation croissante de l’assistance qualifiée à l’accouchement et du taux de césariennes ainsi qu’une diminution des inégalités dans les quatre pays, mais ces tendances étaient déjà en cours avant l’introduction des politiques d’exemption de sorte qu’un changement ne peut pas être attribué seulement à ces dernières. Une réduction significative des charges financières sur les ménages - après l’introduction des politiques - a été trouvée, ce qui suggère que les objectifs de protection financière ont été en partie remplis, au moins à court terme, bien qu’aucun site n’ait réussi à maintenir une exonération totale des coûts ciblés. Dans les quatre pays les politiques sont financées sur le budget national et donc potentiellement pérennes et ont été relativement bien mises en œuvre. En outre, aucune preuve d’effets négatifs sur la qualité technique des soins ou sur les services non ciblés n’a été mise en évidence.

**Conclusions:**

Les politiques ont permis d’atteindre les objectifs de protection financière et probablement les objectifs de santé et d’équité, à un coût pérenne, mais une série de mesures pourrait augmenter leur efficacité. Celles-ci comprennent l’élargissement du paquet de soins exemptés (en particulier pour les pays ciblant uniquement les césariennes), un système de paiement des hôpitaux mieux calibré, des informations plus claires sur les politiques, une meilleure gestion du système de santé local pour faire face à des faiblesses systémiques sous-jacentes, et plus d’attention à la qualité des soins, en particulier pour les nouveau-nés.

## Background

In many African countries the burden of maternal and early neonatal mortality remains extremely high, and delayed access to emergency obstetric care (EmOC) – especially caesarean section – is known to be a major obstacle to progress. In particular, user fees for care are prohibitively expensive for many households and prevent women from seeking professional care when complications during pregnancy or delivery arise [1]. Those that do access care, experience substantial difficulties paying for hospital fees and often resort to selling assets, borrowing from friends or family members or accruing new debt [2]. Removing user fees is a current strategy to increase access to and use of EmOC and has been introduced in many African countries, including Western and Northern Africa as a core policy to reduce maternal and neonatal mortality [3].

While many countries are currently removing user fees for delivery care and especially EmOC, particularly in sub-Saharan Africa (Meessen 2009), the current evidence regarding the impact of this policy is not well developed. In part this is because evaluation designs are not able to capture all the necessary information for policy-makers to make informed decisions. This article brings together findings from complex evaluations by the FEMHealth programme in four countries - Benin, Burkina Faso, Mali and Morocco. It aims to document the costs and impacts of obstetric fee removal and reduction policies in a holistic way. This includes analysis of policy drivers, financing and sustainability, impact on the wider health system as well as effects on quality of care, equity of access and financial protection. Equity is examined across each domain, but with particular emphasis on changing utilisation of care, the financial benefits of the policy for households, and understanding the extent to which the policies address the existing barriers to deliveries and caesareans in the study countries.

## Background on national policies

### Benin

The free caesarean policy was introduced on the 1st April 2009. It applies to caesareans in all public and private hospitals that offer emergency obstetric care (but not to private-for-profit clinics). All women are entitled to benefit and all areas of the country are covered, in theory. Hospitals are reimbursed 100,000 CFA per caesarean to cover: check-up costs before medical intervention, drugs, kits, surgery, blood, and hospitalization for 7 days. The women pay for any costs arising before hospitalization, or any other complication which may arise during the hospitalization. Treatments for other complications which require surgery (such as uterine rupture) are not free.

### Burkina Faso

The national delivery subsidy policy in Burkina Faso started in October 2006 for caesareans and April 2007 for deliveries and complications. It is more complex and comprehensive than the policy in Benin. It operates in all public health and some confessional facilities, and offers an 80 % reduction of fees at health centre, district hospital and referral hospital levels for: caesareans, complicated deliveries and neonatal care. For normal deliveries, there is an 80 % reduction of fees at health centres and district hospitals, but only 60 % at tertiary hospitals (to disincentivise by-passing). The policy covers all of the in-facility costs (hospitalisation, medicine, surgical kit, postoperative care, lab exams, medical act), as well as referral transport costs. There is provision in theory for additional support (full exemptions) for ‘indigents’ (the extreme poor) [4].

### Mali

The free caesarean policy in Mali was introduced in 2005. It is applied nationally to all caesareans in the public sector, and in theory covers all facility-based costs (but not transport). In a three-way partition of costs, families are intended to fund the journey into the health centres, while communities fund the onward referral transport costs, and the state covers the costs of service provision, including hospitalisation, surgery, laboratory tests, and treatment of complications such as pre-eclampsia and ruptured uterus [5].

### Morocco

The free delivery and caesarean policy in Morocco was implemented nationwide from the start of 2009. It covers normal and complicated deliveries in all public facilities (including in theory university hospitals if the woman is referred from a public facility), resuscitation, transport to the appropriate level, and care for mother and newborn as long as they stay in the facility (Ministerial circular of 11 December 2008). Miscarriage/abortion, and ectopic pregnancy are not covered. The Morocco policy is embedded in a wider national strategy for safe motherhood, which includes upgrading of facilities and skills, and also increasing awareness and physical access.

## Methods

As the policies had been introduced nationally, without control areas, the project used a case study design, broadly based on the realist evaluation approach, to understand differential implementation and effects across different sites.

**Fig. 1:**
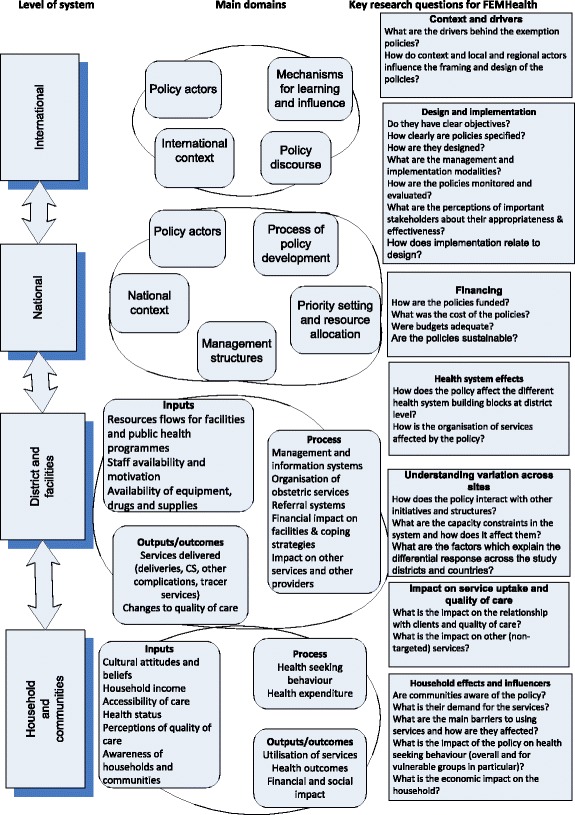
FEMHealth main research questions

Realist evaluation considers that policies work (or not) because actors make particular decisions in response to the resources or opportunities that the policy provides. To understand how the actors introduce change in a certain context, a realist evaluation explores the context in which the policy operates and the mechanisms that drive action. Mechanisms can refer to psychological, social, cultural or organisational drivers that explain why the actors responded to the programme the way they did. Policy triggers mechanisms only in specific context conditions: The context influences the response of the actors: mechanisms (and thus the programmes that trigger them) only work if the context conditions are right - not every programme works identically in every setting. The context-mechanism-outcome (CMO) configuration is the heuristic used in realist analysis, which results in answering the question ‘Did the policy work, how, for whom, why and in which conditions?’. The explanation of how the programme has contributed to the observed results is what realist evaluators call the programme theory. The programme theory is not only the end product of a realist evaluation, it is also its starting point (Fig. 1) [6, 7].

In this study, realist evaluation was applied in two ways. First, we carried out realist case studies of policy adoption and implementation. Second, we formulated programme theories in the form of conceptual frameworks linking all of the components of the evaluation programme at the start, which helped to integrate results into country case studies [8]. A set of core research questions were developed which outlined the main levels, domains and topics that FEMHealth would seek to investigate. These form the broad structure of this article, which flows from an analysis of the drivers behind the policies, their objectives and formulation at the national level to analysis of how they interacted with and impacted on district-level health systems, and, finally, their effects and effectiveness at community and household level (Fig. 1).

The findings presented in this paper are taken from 14 main data collection tools (Table 1), most of which were used in all four study countries.

**Table 1:** Summary of FEMHealth research tools

	Tool and findings section to which it contributes	Level	Key themes	Approach
1	Observation grid in meetings (B-SCALA) *Context and drivers*	Actors at the national, regional and international level	The ways/direction and content of the discussion and presentation of the exemption policy Key concepts: hierarchy, power, evidence, etc.	Participant observation in policy and maternal health meetings
Sample summary: Benin: 1 conference and 10 agency meetings				
2	Interview guide with national and regional actors *Context and drivers; design and implementation*	Actors at the national, regional and international level	Introduction of the policy Perceptions of how the policy was put in place and how it works Actual implementation of the policy compared to official documents Elements of the political context necessary to ensure the policy is implemented and is effective Exchange between national, regional and international actors policy on the policy	Structured discussion with key informants
Number of informants interviewed in the following countries: Benin: 24; Morocco:12; Burkina Faso:23; International: 9				
3	Policy document review *Design and implementation*	National	Review of published reports, analyses, press releases and other documents related to the policy at national level	Thematic analysis
4	Financial flows tracking (FFT) *Financing of policies; facility finances*	National, regional, district, and health facility level	Budgets & expenditure Distribution per region and health services 3. Payment Schedule (and the kits/equipments where necessary) Consistency with the recorded activities Consistency and adequacy of funds arriving at the health facilities	A structured collection and analysis of secondary data
Sites: Benin: national level; 6 regions; 5 districts; 7 hospitals Burkina Faso: national level; 5 regions; 6 districts; 12 hospitals (1 CHU, 2 CHR, 4 CMA, 6 CSPS) Morocco: national level; 6 districts; 8 hospitals (2 CHU, 2 CHR, 4 CHP) Mali: national level; 4 regions; 8 hospitals				
5	Costing *Financing of policies; facility finances*	Health facilities	Unit cost of production of key maternal health services: normal deliveries, complicated deliveries, caesarean sections, antenatal care, postnatal care	Based on interviews and a extraction of information from sample of medical records
Sample: Benin: 7 hospitals in 5 districts; 1050 cases Burkina Faso: 6 districts; 6 hospitals (4 CMA, 2 CHR); 443 cases Morocco: No Costing tool Mali:4 CHR; 4 HD; 2 CSREF; 2691 cases				
6	Exit interviews (EI) *Household-level effects; quality of care*	Women who had a delivery, their husband or relatives who accompanied them at the hospital	Costs for a given delivery inside and outside hospitals Expenditure as a percentage of household consumption Healthcare seeking behaviour Access to health facilities Perceptions of quality of care	Structured questionnaire
Benin: 663 women in total interviewed; 294 with a caesarean; 294 women with a complicated delivery; 81 women with normal delivery Burkina Faso:1609 women in total; 818 with a caesarean; 462 with complications; 316 with a normal delivery Morocco: 973 women in total; 423 with complications; 442 with caesareans; 108 with normal deliveries Mali: 589 women in total; 30 complicated deliveries; 345 caesareans; 188 normal deliveries; 26 without assistance/home delivery				
7	Health worker survey (HWIS) *Effects on human resources*	Health workers	Health workers and their workload Working hours Sources of income Motivation at the workplace Changes in the above factors, associated with the policy Perceptions of the policy	Structured questionnaire (with some open questions)
Sample: Benin: 190 health workers; Burkina Faso: 130 health workers; Morocco: 187 health workers; Mali: 176 health workers				
8	The Policy implementation assessment (POLIAS) *Design and implementation*	District Hospitals	The start of the implementation of the policy The service package covered by the policy The proportion of facilities offering the service package free of charge and on a permanent basis The actual geographical coverage	Structured discussion with key informants; Documentary review (for triangulation purposes); Routine data extraction
Benin: 5 districts and 7 hospitals; Burkina Faso: 6 districts and 6 hospitals; Mali: 8 districts and 8 hospitals; Morocco: 6 districts and 6 hospitals				
9	Policy Effects Mapping study (POEM) *Effects on health systems*	District Health management team Management team at the hospital Health workers	Governance Provision of care Human Resources Financial resources Drugs and equipment Health Information System Patients & the community	Interviews with key informants Documentary review Routine data extraction Check-list/observation
Benin: 85 interviews in 4 districts hospital, 2 private hospital,1 departmental hospital, 10 health centres Burkina Faso: 57 interviews in 4 districts hospitals and 2 regional hospital and 12 health centres Mali: 84 interviews in 4 regional hospitals, 4 district hospital and 16 health centres. Morocco: 110 interviews in 5 districts hospital, 2 regional hospitals, 2 university hospital, 12 health centres				
10	Realist case studies *Factors behind differential implementation*	Districts hospitals	Actual implementation of the policy compared to official documents Perceptions of managers on the challenges posed by the new policy Mechanisms that explain the ownership and the implementation of policy at the operational level. Contextual elements necessary for the policy to be effective	Interviews with key informants Documentary review Routine data extraction Using data from other tools for triangulation.
2 districts/country (excluding Mali); Benin: interviews from POEM; Burkina : interviews from POEM + 16 extra interviews to complete the analysis; Morocco: interviews from POEM				
11	Quantitative instrument on near-miss, caesarean sections and the quality of care *Impact on quality of care*	Women and newborns	The outcome of hospitalisation The demographic characteristics The reproductive history The causes of complications The near-miss definitions for women and newborns The indications for caesarean section Delays in receiving care Quality of care for caesarean section Quality of care for all women	Medical records and records of admitted women in the maternity ward (normal deliveries, near-miss, caesarean sections)
Benin: 3361 deliveries; Burkina Faso: 1752 deliveries; Morocco: 3134 deliveries; Mali: 6386 deliveries				
12	Quantitative analysis of secondary data *Impact on utilisation; inequities of access*	National	Utilisation of facility delivery care Trends in caesarean section rates Equity of access	Segmented regression analysis of data from routine annual statistics and nationally-representative household survey data The model was specified as: *Y*_*t*_ = *β*_*0*_ + *β*_*1*_ *time + *β*_*2*_ *policy + *β*_*3*_ *postslope + *ε*_*t*_ Where *Y*_*t*_ is the outcome variable (either facility delivery or caesarean delivery) at time *t*; *time* is a continuous variable; *policy* is a dummy variable indicating whether or not the policy has been implemented at time *t*; and *postslope* is coded 0 up to the last point before the introduction of the policy and coded sequentially from 1 thereafter Based on recommendations by [22]
Benin: Demographic and Health Survey data for 1993–2011 (*n* = 36,375) Burkina Faso: routine data published by the Ministry of Health for 1992, 1998 2000–2010; Demographic and Health Survey data for 1988–2010 (*n* = 36,836) Mali: Demographic and Health Survey data for 1993–2013 (*n* = 43,952) Morocco: routine data published by the Ministry of Health for 1997–2011; Demographic and Health Survey data for 1987–1992, 1998–2011 (*n* = 16,679) Missing data points in Figs. 2, 3, 4, 5, 6 and 7 are due to lack of coverage due to gaps between DHS.				
13	Observation guide in health facilities *Impact on quality of care; other household-level effects*	Health facilities	Quality of care for all women Quality of care for caesarean sections Delays in receiving care Communication between staff, patients and their carers Resources (human, materials, etc.) Costs and payments for services	Participant observations in hospitals
Benin : 4 weeks’ observation in 2 hospitals; Morocco :3 weeks’ observation in 2 sites				
14	Interview guide with women *Impact on quality of care; other household-level effects*	Health facilities/community (women)	Perceptions of quality of care Perceptions of costs related to hospital delivery Awareness of free care	Structured discussion with women after they return home
Benin: 44 caesareans; 9 Near Miss; 9 “normal” deliveries; Morocco: 30 Near Miss				

Within each country, districts were classified using four variables: skilled attendance at delivery, caesarean rate, poverty rate, and geographical access. The project used a hierarchical cluster method to choose 4-6 districts per country from different homogenous groups. This article draws from a range of research papers and country reports to provide comparative insights (further details can be found at www.abdn.ac.uk/femhealth, which also has reports on the individual country findings).

All protocols received in-country ethical approval – in October 2011 in Burkina Faso, January 2012 in Morocco, February 2012 in Benin, and July 2012 in Mali. In addition to the approval from the ethics committee, administrative authorization was requested and obtained from the regional health departments, districts, hospitals and at national level in all four countries. The component tools supported by thematic work teams were also approved by the ethics committees at the LSHTM and ITM.

## Results

### Context and drivers behind policies

One of the FEMHealth research aims was to understand the origins of the policies in this region – why had so many countries adopted similar policies over a short period? What were the drivers behind this and how far had they been influenced by one another and by international actors? These were addressed mainly through research tools 1 and 2.

One set of drivers related to the recognition by decision-makers that socio-economic factors were behind low overall skilled birth attendance rates in Mali, Burkina Faso and Morocco and large inequalities in all four countries. In the early-mid 2000s, around the time of their policy discussions and implementation, Benin, Burkina Faso and Mali had slightly lower maternal mortality ratios compared to the sub-Saharan country average of 680 per 100,000 in 2005 (Table 2) [9]. All of these ratios had shown declines in the ten years before the policies were adopted, however not at a rate seen to be able to reach the targets set by the Millennium Development Goals (MDGs). The Morocco maternal mortality ratio at the time of the introduction of the policy was considerably lower - 227/100,000 overall (rising to 267/100,000 in rural areas).

**Table 2:** Summary of maternal and neonatal health indicators by study country at start of policies

	Benin	Burkina Faso	Mali	Morocco
Sources	DHS 2006	DHS 2003	DHS 2006	DHS 2003–2004
Maternal Mortality ratio	397/100,000	484/100,000 (DHS 1996)	464/100,000	227/100,000
Neonatal Mortality rate	32/1000	31/1000	46/1000	27/1000
% Skilled birth attendance rate	78 %	57 %	49 %	63 %
Coverage of antenatal care (at least one visit)	88 %	73 %	70 %	68 %
Coverage of antenatal care (at least four visits)	61 %	18 %	35 %	31 %
% Caesarean deliveries	4 %	0.7 %	1.6 %	5.4 %

The caesarean section rates at the time of policy implementation in the three sub-Saharan countries were below the WHO/UNICEF recommendations of 5–15 % for these settings. Wealthier women were more likely to have caesarean sections than poorer women [10]. In Morocco 16 % of women in the wealthiest quintile had caesarean sections, while that percentage already dropped to only 1.5 % among women in the second wealthiest quintile [11] (Ministerial circular of 11 December 2008). Facility delivery rates were notable across these countries in terms of a rural/urban divide – women in rural areas tending less frequently to attend facilities to deliver than those in urban areas. In terms of overall rates for facility births, Benin had the highest rate at 78 % in 2006, with Morocco following at 63 % in 2003–2004, Burkina Faso at 57 % in 2003 and Mali at 49 % in 2006. All countries showed a higher rate of women attending ante-natal care consultation at a health facility than delivering in facilities.

Our initial hypothesis that international actors might have been behind the proliferation of related policies in the West Africa region was not sustained: interviews suggested that while international influences have been important in shaping the global climate which permeates the four case study countries, the decision-making and elaboration of the policies were dominated by local factors. International actors may have lost some credibility through changes in stance on issues like user fees, with strong but changing messages over the past two decades.

*“… I think word was getting out, around Africa, that this policy [charging user fees] was mad… To remove fees was good for the health sector and also brought big political benefits. And I would say this whole thing has been a politically driven process rather than a technical one, and that remains to this day. […] I think a lot of developing country governments now are rather sceptical about the advice they’re getting from development agencies, because in many respects, we forced them into this in the first place, so for us to now turn around and say ‘oh no, you shouldn’t do that, you should remove them’ – I think that people are sceptical about a lot of the advice that we are providing.” –* Consultant with international agencies, global level (GL1)

West African countries that were once the heartland of the Bamako Initiative (a cost-sharing initiative promoted by international agencies since the 1980s) have been amongst the most active in taking up selective exemptions (which were seen as more acceptable and affordable than broader approaches to fee removal). The emphasis on the MDGs and, now, on universal health coverage have influenced the underlying discussions, and these free care policies have to be seen in a context of proliferating exemptions in the countries and region for many different vulnerable groups and priority services, though variably implemented. Shame at performing less well in relation to neighbours was highlighted as a driving factor for Morocco. Personal political leadership was seen as critical in all contexts to enabling the policies to be realised – particularly for setting out a vision, and mobilising funds and support. Evidence, while it was marshalled quite thoroughly in two countries (Morocco and Burkina Faso), was used more to assist with planning implementation details than in propelling the original policy adoption itself. Affordability was one factor behind the narrowing down of services in Benin.

### Design and Implementation of the policies

Table 3 summarises the services which were included in each package and Table 4 the kinds of costs which were covered.

**Table 3:**
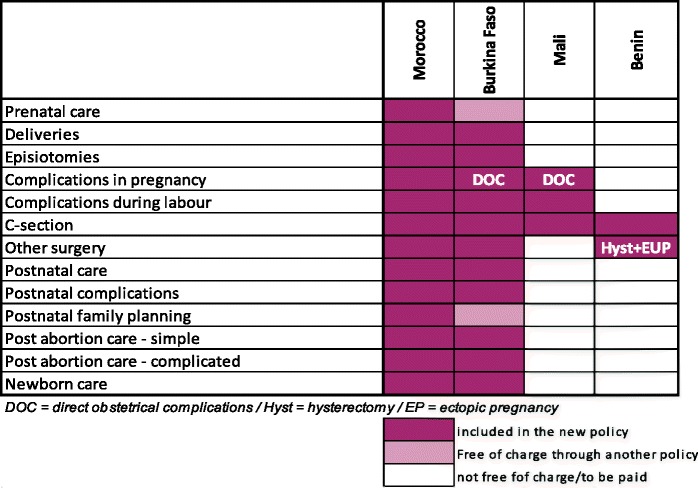
Package of care covered by the exemption policies, all countries

Source: document review and key informant interviews

**Table 4:**
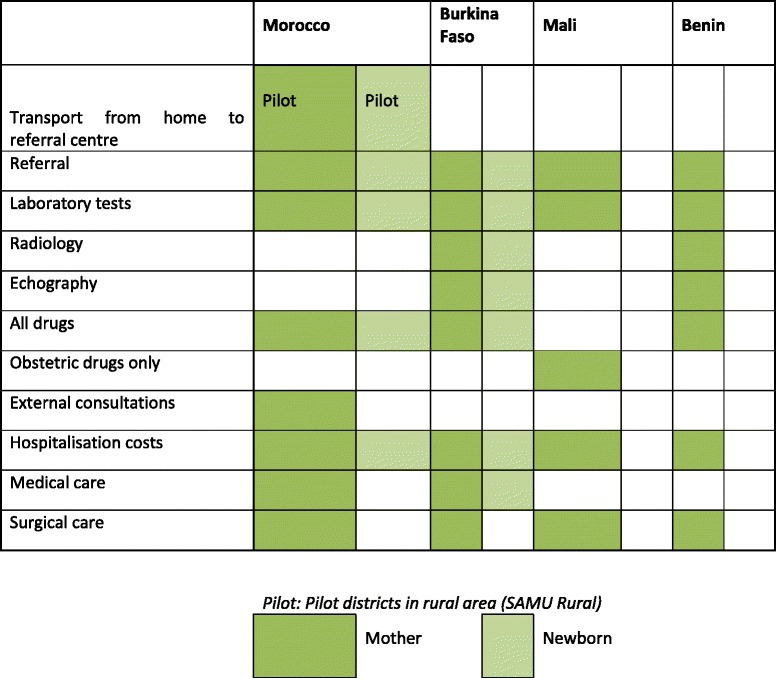
Type of costs covered per target group in the four FEMHealth countries

Source: document review and key informant interviews

All medical costs associated with the target services within hospitals (and health centres in Burkina) were included in principle in the package. None of the countries covered transport to the first level facility, but all claimed to cover onward referral transport, though in practice managers acknowledged that patients often paid. For Morocco, food within the hospital was covered, but this was not the case for the other countries. Within hospitals in each country, however, the package was interpreted differently at the time of the study. Of 14 items which were mandated or implied by the policy in Benin, the focal hospitals provided between 4 and 11 completely free, according to key informant interviews with managers. For Morocco, 4–5 out of 6 items were provided fully without charge within our study hospitals. For Burkina Faso, few understood that newborn care or post-abortion care were a part of the package, and there was no free care provided for these services.

Management arrangements also varied: most policies were managed by a national committee but in the case of Benin, a dedicated autonomous agency was established to manage the policy. These differences are reflected in some of the research findings: for example, the centralised model adopted by Benin may explain why there were no discrepancies or major delays in reimbursement flows to facilities. However, there were also downsides, in terms of a lack of involvement of the health zone in managing and monitoring the policy (the chain of command went straight from the agency to the hospitals, without involving district managers).

Supervision and evaluation was planned as a part of these groups’ work but tended to be carried out less frequently than envisioned (for example, in Mali the twice-yearly supervision tended to be carried out once a year).

### Financing

In all four countries, the policy is financed almost entirely by the state, with a notable absence of direct donor financial support, and indirect donor support for the sector ranging from 0.5 % of public health spending in Morocco to 36 % in Burkina Faso.

The scale of investment was hugely different across the countries, with Morocco spending in the region of 24.5 million Euros on its overall action plan in 2011, compared to 3.2 million Euros in Benin, and 415,000 Euros in Burkina Faso. Per delivery covered that equated to 1.3 Euros for all kinds of deliveries (Burkina), 152 Euros per caesarean (Benin), and 797 Euros for all deliveries combined (Morocco). Clearly, these differing scales of investment should shape our expectation of results.

As a proportion of public health expenditure in 2011, the policies absorbed around 2.5 % in Morocco, 3 % in Benin and 3.5 % in Burkina Faso – not insignificant but all potentially sustainable, if the policies are seen as effective.

The three sub-Saharan African countries reimburse retrospectively according to the number of services provided, using fixed payments per caesarean in Mali and Benin, and reimbursement of actual variable costs in Burkina Faso. For Morocco, hospital budgets have been boosted to reflect rough estimates of revenue likely to be lost due to the withdrawal of fees for deliveries, but these are set in advance, are not linked to services delivered and have not increased over time (indeed all subsidies for hospitals were unpaid in 2011). Additional investments in staffing and improved access and care were made in Morocco, which was not the case for any of the other countries, where the budget for the policies just covers the cost of facility reimbursements. Management costs were limited in three countries. Only Benin had an agency set up to operate the policy; in the other three, the management was added to the existing workload in the Ministry of Health. These features are reflected in some of the findings on implementation and effectiveness.

The payment systems also create specific incentives. For Burkina Faso, decision to shift from fixed payments per delivery to payment of actual costs means that there is no incentive to over-provide or to skimp on quality, but on the other hand, there is no surplus to reinvest (the costs are just the variable ones, such as drugs and supplies, so there is no contribution to general facility running costs). In addition, the workload implied by billing by item is considerable, which is why this factor caused discontent amongst the staff in Burkina Faso, more than in any other country. By contrast, the fixed tariff in Benin was found to overpay caesareans at all types of hospital (relative to actual production costs but also relative to previous payments from users). On the positive side, examples of managers reinvesting the surplus in improving overall services were found in some sites in Benin. On the negative side, there was a perception by some key informants in Benin that staff were sometimes too eager to do a caesarean, rather than letting women try for a normal delivery. In addition, despite the generous payments, some hospitals continued to make women pay for items which should have been free (see below).

### Health systems effects

#### Human resources

The only country to accompany its policy with a significant increase in staff was Morocco, which increased the deployment of midwives and hospital specialists as part of its overall action plan. For other countries, there was no evidence of increased numbers of staff in any large measure, though in Benin, the additional resources provided by the policy allowed some local changes to improve staffing. Table 5 shows that even after the introduction of the fee exemption policies, working hours remained reasonable across the countries, and productivity relatively low, with around one patient seen per hour worked (in Mali and Burkina Faso). While higher median working hours are reported in Morocco, the outputs are not necessarily higher – indeed for some categories, such as doctors, even fewer patients seen are reported. WHO’s Making Pregnancy Safer model suggested that 1 doctor is required for around 1000 births, to provide emergency intervention where there are complications before, during and after delivery, while a midwife can provide care for 175 births per year. If these self-reported figures are accurate and are scaled up to get annual estimates, midwives are over-committed in all four countries, while doctors are within norm in all countries. Analysis of routine data for deliveries, divided by staff quota, produces lower ratios. In part, this may be due to several staff contributing in different ways to a single delivery and working on many different services, but may also reflect over-reporting by staff.

**Table 5:** Average number of hours worked, patients seen and deliveries done weekly per staff member, by professional category, across countries

Country	Professional category	No. of hours worked (incl. on call)	No. of patients seen	No. of deliveries performed
Burkina Faso	Doctors	42	45	3
	Midwives	44	44	6
	Nurses	46	71	6
Morocco	Doctors	70	36	0
	Midwives	48	74	25
	Nurses	40	20	8
Benin	Doctors	48	28	6
	Midwives	48	26	12
	Nurses	48	16	4
Mali	Doctors	40	33	4
	Midwives	40	25	6
	Nurses	36	27	3

*Source: Health worker survey*

However, workload (patients seen and deliveries attended) are reported to have increased for three out of four of the countries (Mali being the exception). In Burkina Faso, the administrative workload imposed by the policy was a particular concern. The consensus was that the policies have not affected their remuneration (as there are no direct financial incentives linked to it for any of the staff in any of the countries).

Across all countries the majority of staff surveyed felt positive about the wider effects of the policy, believing that the policy has increased access to skilled birth attendance, has benefited the poor, and has improved the quality of care, including through perceived improvements to drugs and supplies. This also impacts positively on health worker working conditions and satisfaction in some countries, like Burkina Faso and Mali.

#### Facility finances

In Morocco, despite the increased budget to accompany the implementation of the free caesarean and delivery policy, the general revenues of the health facilities have not been affected positively. In 2011 and during the first half of 2012, no financial resources were provided at all. This has affected the financial reserves of the health facilities that are still implementing the gratuity policy. For Benin, the costing revealed that the current tariff is beneficial to hospitals, paying considerably more than it costs to provide the service, though the surplus varies by type of hospital. In addition, payments have been regular. For Mali, the calculus is different, with the reimbursements not fully covering costs of provision. In Burkina Faso, payments are often delayed due to the difficulty of justifying expenditures and do not include any operating costs beyond the direct inputs needed for the service.

It might be expected that extra costs would be levied from users in countries where facilities are poorly rewarded for providing care, however analysis of household payments does not support this. In absolute terms, women reported paying the largest amount overall for caesareans in Benin (60 Euros on average), where the payment for the policy is most generous. This suggests that organisational culture and other factors play a role in the levying of additional payments from users, rather than actual financial need from the facility perspective.

#### IT systems

There has been no broad effect of the policies on health information systems, either positive or negative. In Benin, there has been a policy-related improvement in obstetric information gathering, but this has not cascaded into other areas of health information. In Burkina Faso, a specific system of collecting information on the policy was put into place. Unfortunately the specific software crashed several times, forcing the district managerial teams to re-code all the individual forms of the beneficiaries dating back to the beginning of the policy implementation. These IT bugs created a lot of frustration and delays in reimbursement. As for Morocco, the wider information system is unchanged and is generally seen as onerous for health staff.

#### Drugs and supplies

All four countries used kits to support the implementation of the policy, with varying results. The policy in Morocco was accompanied by a large increase in kits, which meant that drug supply improved, though in some places, kit numbers were well in excess of need. For Burkina Faso, drug supply also improved, but management of kits sometimes lacked transparency and stock-outs occasionally occurred. In both Morocco and Burkina Faso, equipment was reported by key informants to be lacking, with no provision made to improve working conditions. In Benin, the kit contents were perceived to be too generous at first, leading to misuse, and then too restrictive, leading to costs to users for items not included in the kits. In general, it could be said that attention was paid to providing specific drugs and items needed for obstetric care, but there was no wider investment in the supply system and a continuing focus on the use of kits, which may not be the most efficient method of organising supplies.

#### Management

The policies introduced threats and opportunities for local managers, and examples were found of positive and negative adaptation. Generally, managers had not been much involved in the development of the policy, and in many cases detailed guidance on how to implement it was lacking. Management teams were found to have some leeway to interpret the policy. In some cases, it was found that actors such as directors and specialists used their power position to adapt the policy to their own benefit, e.g. in some study sites of Benin or Morocco, patients were still charged fees, because staff were compensating for the free caesareans by charging something else to the patient. These effects can be moderated or avoided by capable management teams and adequate supervision by programme managers, as seen in other study sites of the same countries, where district health managers and or hospital directors were positively engaged with the policy implementation and the protection of service users.

In most study sites in Benin, Mali and Burkina Faso, the management teams did not have a large absorption capacity to take up the new tasks without additional resources. If the reimbursements were late or inadequate, the implementation halted. In Morocco, this was not the case, as the capacity of the teams and the existing implementation infrastructure in terms of human resources, facilities, and equipment was adequate to take up the new patients.

### Factors behind differential implementation

The analysis of divergent responses of different cadres within our realist evaluation case studies indicated that the adoption of the policy is explained in part by the configuration of autonomy, decision space and motivation of these actors and by organisational, institutional factors and contextual factors.

Different responses were found in different sites within the same country, indicating that beyond policy design and national features, there is an inter-play of local factors that influences whether the policy is blocked, adopted or adapted. In general, the policy was well adopted by the hospital managers. Nurses and midwives in general perceived the policy as a positive one and adopted it by default. Doctors, and especially specialists, were often found to use their power position to implement the policy half-heartedly or to change it to their advantage. At personal level, personal commitment and perception of opportunity were found to play a role, while at organisational level, pressure of local communities, alignment with local needs and enforcement by hierarchy were among the factors that facilitated adoption and positive adaptation of the policy. The role of ensuring public accountability, in particular, was found to be underdeveloped or even not mandated clearly. In any case, lack of effective stewardship allowed faulty implementation processes to continue in many of the sites.

The study confirmed that to implement the policy as intended, managers require adequate margins of freedom in terms of health workforce management, but also reliable supply of drugs and equipment, regular reimbursements and clear operational guidelines. In addition, our studies found that at least as important is a strong public service ethos (reflected in stewardship), which contributes to the use of the above conducive decision spaces and resources in the interest of the public. Finally, we found that health staff are more likely to positively adapt the policy to the local context if they are given supportive supervision, either by the regional directorate or by the agency responsible for the policy.

### Impact on utilisation

**Fig. 2:**
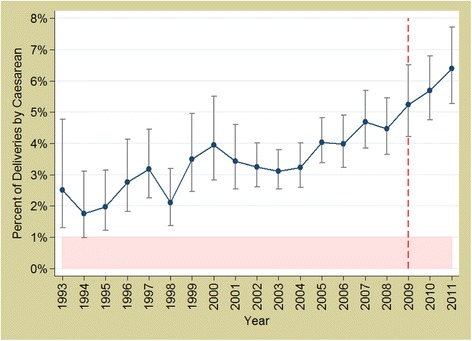
Trends in caesarean section rates in Benin 1993 to 2011. *Source: DHS data; red, dashed line represents the policy (2009)*

In Benin, there was a positive trend in caesarean section rates between 1993 to 2011 (*p* < 0.001), but we found no evidence that the implementation of the exemption policy in 2009 significantly increased utilisation rates over and above the existing secular trend (*p* = 0.7331) (Fig. 2).

**Fig. 3:**
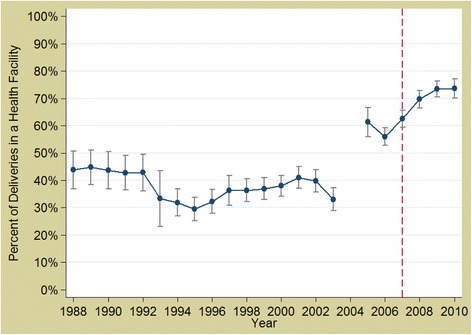
Trends in facility delivery rates in Burkina Faso 1988 to 2010. *Source: DHS data; red, dashed line represents the policy (2007)*

**Fig. 4:**
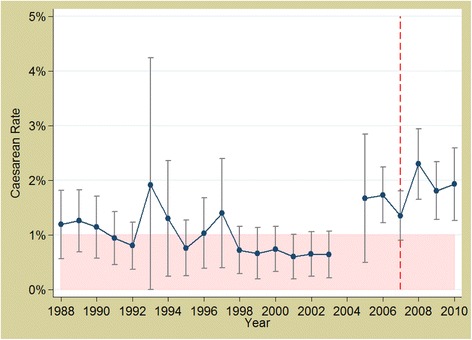
Trends in caesarean section rates in Burkina Faso 1988 to 2010. *Source: DHS data; red, dashed line represents the policy (2007)*

In Burkina Faso, the overall rate was increasing in absolute terms, it was doing so less rapidly post-2007 (Fig. 3), between 2002 and 2007 there was a 12 % relative increase each year, whereas between 2007 and 2010 there was a 6 % relative annual increase (*p* < 0.0001). For caesareans, there was no evidence of a change in either direction (0.4977) (Fig. 4).

**Fig. 5:**
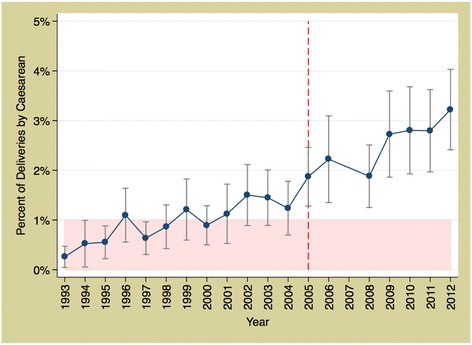
Trends in caesarean section rates in Mali 1993 to 2012. *Source: DHS data; red, dashed line represents the policy (2005)*

In Mali, there was a significant positive trend in caesarean section rates throughout the period 1993 to 2012 (*p* < 0.001) but we found no evidence that the implementation of the exemption policy in 2005 significantly increased utilisation over and above the secular trend (*p* = 0.4991) (Fig. 5).

**Fig. 6:**
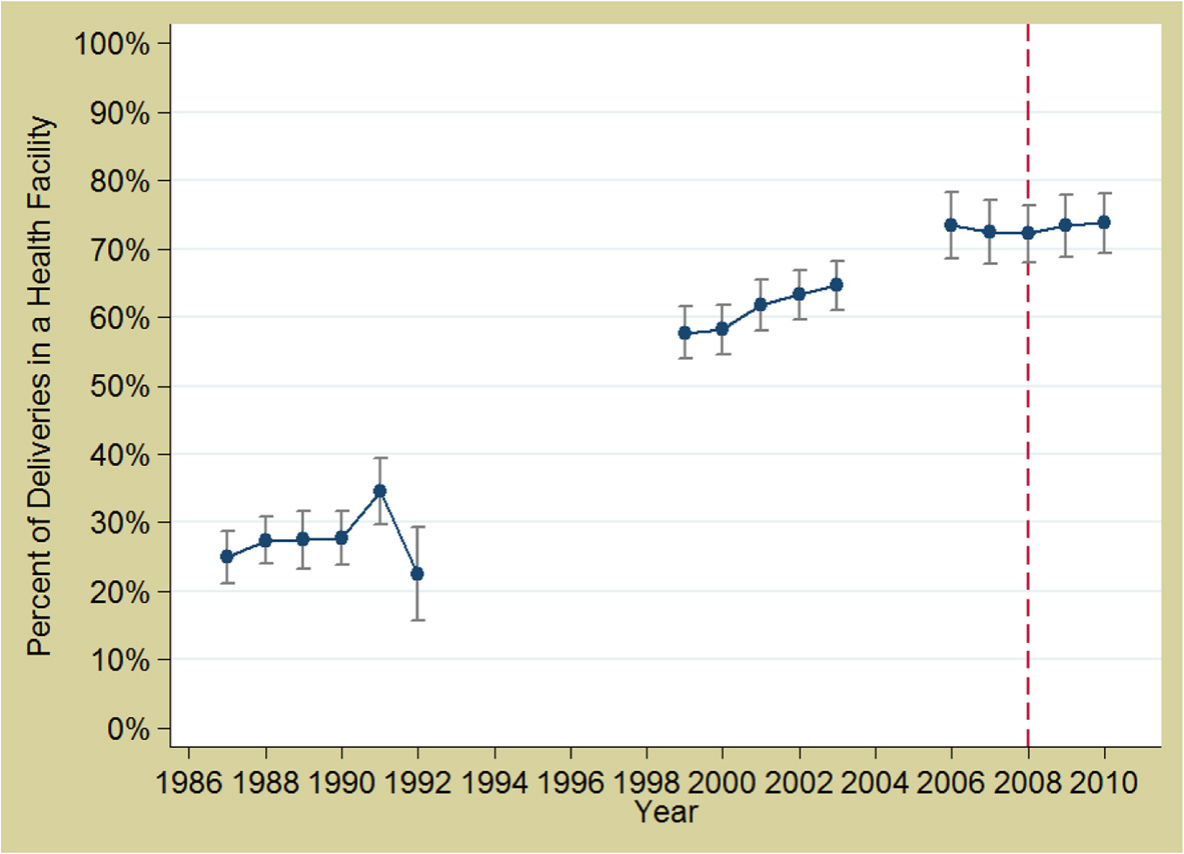
Trends in facility delivery rates in Morocco 1987 to 2010. *Source: DHS data; red, dashed line represents the policy (2008)*

**Fig. 7:**
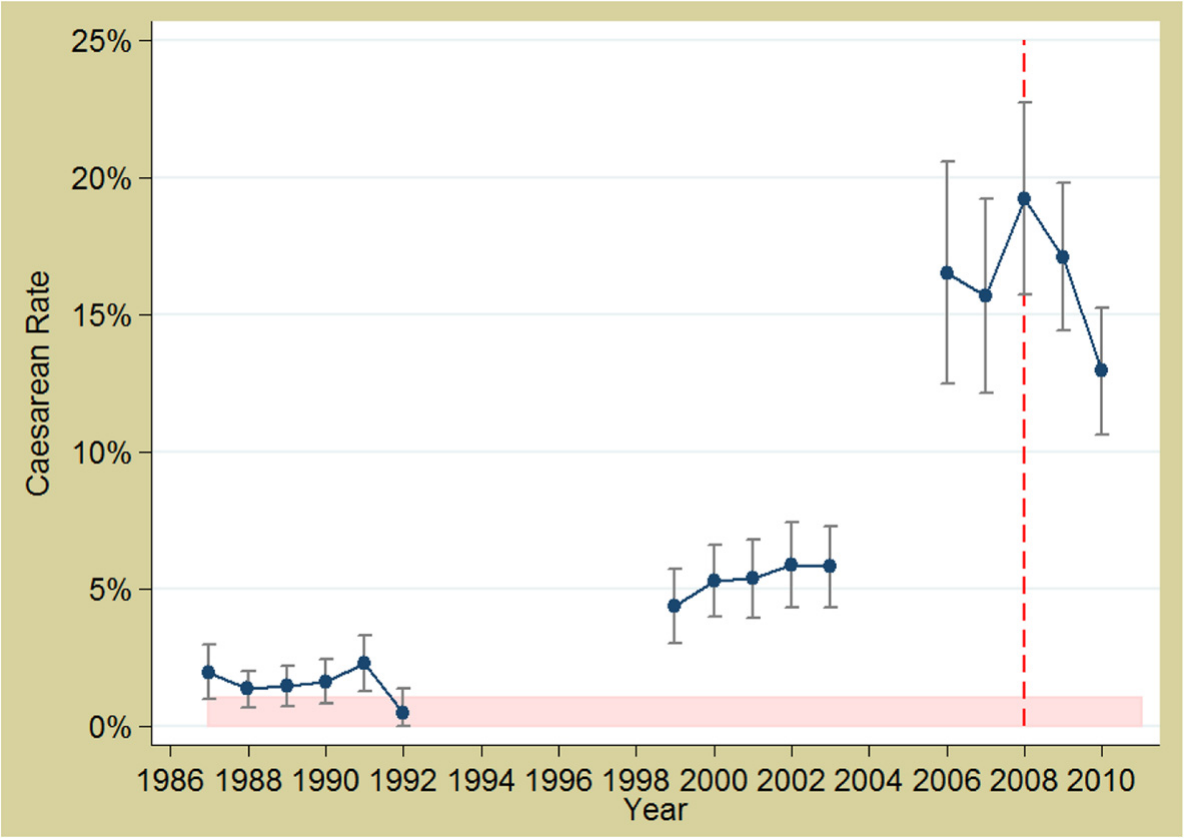
Trends in caesarean section rates in Morocco 1987 to 2010. *Source: DHS data; red, dashed line represents the policy (2009)*

For Morocco, there was no evidence of a change post-2008 in facility delivery rates (*p* = 0.2972) (Fig. 6). There was no evidence that the implementation of the policy increased caesarean rates in Morocco (*p* = 0.6539) (Fig. 7).

It is evident overall that countries have made progress over the past 15–20 years, and at best these policies may have contributed to maintaining the momentum, but there is no statistical evidence of that as yet. It is almost certainly too early to tell, as we have only 2–3 post-policy data points in each country, and the varying implementation documented by the research also underlines the need to be cautious about assuming immediate effectiveness of policies.

Furthermore, the Morocco caesarean section rate is at a level where further increase would be unlikely to lead to substantial additional reductions in maternal mortality, and where there are rising concerns about excess and unnecessary caesareans among certain groups.

### Impact on other (untargeted) services

The research looked in a systematic way for the impact of the policies on untargeted services (such as general medicine and paediatric services), in order to capture any unintended positive or negative effects, but found no major effects. Some positive effects of additional resources introduced by the policies were documented (e.g. in Morocco and Benin), as well as from wider utilisation uptake in Burkina Faso, linked to the policy. On the negative side, some examples were found where the policy encouraged resources (such as staff) to move from untargeted to targeted services. More significantly, in Benin, there was some evidence from trend analysis in some sites of supply-induced demand for caesarean sections, at the expense of normal deliveries. Trend analysis of provision of general medicine and paediatric services in all countries and sites did not reveal any systematic evidence of distortions linked to the exemption policies.

### Impact on quality of care

Women’s perceptions of the overall quality of the services, as reported in exit interviews, were generally high or very high, and did not correlate well with technical quality of care scores (being highest in Burkina Faso, where technical scores were lowest). The quality of neonatal care, measured by the number of omissions in routine neonatal procedures, was very poor in some hospitals in Benin and Burkina Faso, and generally poorer than the quality of maternity care. Median delays in receiving caesarean sections were above the expected threshold of 1 h in most hospitals (except in Morocco) and were the highest in Benin hospitals, even though the policy was designed to facilitate access to life saving emergency surgery. Hospitals in Morocco performed consistently better than hospitals in the other countries.

In observations and interviews in Benin and Morocco, incidents of poor communication between health workers and clients were noted, including lack of informed consent for surgical care and poor bedside manners. In Benin, while poor interpersonal relationships in maternity wards had been documented in previous studies, in this project these were attributed by several respondents to perverse effects of the free caesarean policy.

Hospitals are still receiving many cases of near-miss, particularly maternal near-miss, with hospital incidence ranging from less than 2 % in Morocco to 14 % in Benin. As cases of maternal near-miss are women who nearly died and were saved *in extremis*, there is still a lot of progress to be made in the organization of the health services in order to reduce the burden of several morbidities and mortality in the focus countries. A high burden of perinatal mortality and neonatal near miss was also observed across all facilities.

Our key hypotheses included that hospitals/districts with lower user fees cost may register shorter delays and fewer adverse events because women may arrive earlier in facilities; but that on the other hand, an increase in volume of patients, if not met with an increase in human resources, might lead to a deterioration of the quality of maternity and neonatal care. In Burkina Faso, the hospitals where the costs paid by households were the lowest were also the hospitals with the best technical quality of care as measured by their low omission scores, smaller delays and their low case fatality rate for severe complications. However, limited relationships exist between the omission and implementation score in Benin, implying that quality of the care provided was affected by many factors, which may be quite independent from policies designed to increased access.

### Other household-level effects and influencers

Awareness of the policies was relatively low at the time of the study, ranging from 20 % amongst women who had delivered in Benin to 53 % in Mali (but much lower in some districts). As these interviews were conducted with women who had used services and had already delivered, wider awareness can be assumed to have been even lower at the time. Detailed knowledge of entitlements was very low. Clearly, if policies are to influence care-seeking a greater communication effort is needed, especially for poorer and more remote women (awareness tended to rise for higher quintiles).

#### Delay in seeking care and health seeking behaviour

In general, median reported delays in deciding to leave home, arriving at facilities and being seen were acceptable, although there was a large variation between sites and by type of delivery, and a wide range within the responses in general. The median delay in leaving the house was rather high in Benin. In addition, it is important to remember that those interviewed represent those who were able to access care, and these are often not the most disadvantaged women. Perceptions of need, the availability of transport and the availability of the key decision-maker (who varied by site) emerge as significant.

In-depth interviews with users suggest an appreciation of the policy and that the policy did address some of the key barriers to access. However, it did not necessarily change health seeking behaviour. In Morocco, interviews showed that the choice of location for deliveries was made largely according to expected comfort, care and monitoring (for example, at home), and reassessed in cases where outside help was decided to be necessary as a matter of urgency. Transport was a key barrier. At the hospital, the absence of a doctor, the gaps in surveillance, inadequate resources, and tedious negotiation process to receive the desired care were all aspects known and anticipated by women when considering their recourse to care.

In Benin, there was also no evidence from the interviews or observations that women modified their decision to seek out skilled professionals at birth due to the policy. While the policy was appreciated and the costs of caesarean sections were considered to be more affordable than in the past, the policy does not erase the fear of the caesarean section as a medical procedure and the threat of loss of life. Decisions about where to give birth were based on perceived medical need, hospital reputation, convenience of access and past delivery experiences.

#### Inequities of access

In all three countries for which there was recent household survey data (i.e. excluding Mali), the relative inequity between the poorest and the richest had declined over time (in that there have been bigger gains among the poorest). The policy may have contributed to this but this is a longer term trend, and one which followed to some extent from the fact that richer women already had high coverage. In all three countries there remains substantial inequity in utilisation of care.

In all three countries there is a larger relative difference in utilisation of caesareans, compared to institutional deliveries Inequity is however difficult to interpret with caesareans because the ideal is not 100 % use, so the relative gap is not always meaningful. For example, in Morocco we have clear evidence of unnecessary caesareans taking place among the richest as the rate is over 25 % [12]. Without data on the reasons for the caesarean sections it is hard to assess if lifesaving needs are being met or not. Burkina Faso’s situation is quite different: the caesarean rate is only just reaching above 5 %, even in the wealthiest group.

In Morocco, the top quintile and to some extent the fourth quintile make substantial and growing use of the private sector for deliveries. This alleviates the public finance burden of the policy as the rich self-select out of its use to some extent.

#### Financial impact for households

In relation to the amounts that should have been paid by households under the policies, we found that they are paying excessive amounts in all countries, though the excess payments are relatively low for Morocco (2 % of their total payment for caesareans and 6 % for normal deliveries), which indicates a relatively effective implementation of the policy (Tables 6 and 7). By contrast, in Mali, 49 % of the household payment is excessive. Intermediate proportions of 13 % (for Benin) and 17 % (for Burkina) were found for caesarean sections. The absolute amounts paid in Benin were higher than for Burkina, as the overall payments were higher.

**Table 6:** Total payment per caesarean section (in Euros) and % as proportion of household monthly expenditure

	HH Expenditure per month	Excess amount paid per CS	Total payment for CS	Excess as % of total payment	Payment per CS as % of median household expenditure
Burkina Faso	61.89	6.25	36	17	58
Morocco	178.26	1.44	63.2	2	35
Benin	90.89	8.74	64.54	14	71
Mali	100.90	23.92	49.2	49	49

*Source: Exit interviews*

**Table 7:** Total Payment per normal delivery in Burkina Faso and Morocco (in Euros) and % as proportion of monthly household expenditure

	HH Expenditure per month (EUR)	Excess amount paid per ND (EUR)	Total payment for ND (EUR)	Excess as % of total payment	Payment per normal delivery as % median HH expenditure
Burkina Faso	61.89	0.61	16.23	4	26
Morocco	178.26	3.12	48.73	6	27

*Source: Exit interviews*

Note: Excess refers to payments for items which are explicitly included in the exempted package of care (or over the basic tariff, in the case of Burkina Faso)

Total payment refers to the total cost of a caesarean (costs paid inside the hospital including gifts to personnel, costs of prescribed drugs bought either at the hospital or private pharmacy and the costs of carers)

Patterns of payment across quintiles and across rural and urban areas varied (Table 8). Large proportions of the households in all settings and quintiles incurred catastrophic costs, even under the current policies (and a higher proportion in rural than urban areas in all four countries).

**Table 8:** Total expenditure per delivery^a^ as % of household expenditure, by rural/urban location and by quintile

	Quintile 1		Quintile 2		Quintile 3		Quintile 4		Quintile 5		Average payment (Euros)
	Urban	Rural	Urban	Rural	Urban	Rural	Urban	Rural	Urban	Rural	
Burkina Faso	56	71	115	78	35	124	40	60	23	60	50
Morocco	52	54	44	58	35	32	31	36	23	69	49
Mali	147	64	49	50	89	62	32	37	34	52	50
Benin	139	170	-	124	124	163	106	170	133	101	83

*Source: Exit Interview*

^a^Total payment refers to the total cost of delivery (costs paid inside the hospital incl. gifts to personnel, costs of prescribed drugs bought either at the hospital or private pharmacy, the costs of carers and transport costs)

Certain costs, including transport, the expenses of companions, care of newborn (in some countries), tipping of health workers and supplementary drugs remain a burden. Lack of clarity on charging can also reduce the predictability of costs and cause anger and confusion for women and their families.

Between 0 and 35 % (depending on the site) of households surveyed had been unable to make the requisite payments, with those in lower quintiles more likely to report this than in higher. In general households coped by using savings and getting help from family and friends, but some had to sell productive assets such as land to cover the bills. Very few had health insurance membership and in general, health insurance was not a protective mechanism for most households, as it is pro-rich in its distribution.

Looking at the difference between recorded payments prior to the policies and average payments now, households have made a substantial financial gain. In Burkina Faso, there was a reduction of 71 % for deliveries of all kinds. In Morocco, the gain was lower for normal deliveries (62 %), compared to 92 % for caesareans. The estimated saving for caesareans in Benin was in the region of 74 %, compared to 78 % for Mali.

## Discussion and conclusions

The main overall study limitations were an absence of baseline indicators for many variables, which limited our ability to trace policy impact directly; gaps in secondary data for a number of areas; and civil conflict in Mali, which meant that only some of the research tools could be completed there. In order to study effects, FEMHealth used previous studies, where available, and time trend analysis [12, 13]. We also used cross-sectional analysis to understand how differences in outcomes might link to differences in implementation of the policies. Our overall approach was to construct a plausible narrative of policy introduction, implementation and effects, based on a triangulation of different sources and methods.

The policies in Burkina Faso, Benin, Mali and Morocco were strong national initiatives, which aimed to improve maternal health and to increase access to obstetric care. On one level, the evaluation produces inconclusive results: we observe positive trends in relation to skilled birth attendance and caesarean sections and a narrowing of inequalities in all three countries for which recent data is available but cannot attribute these to the policies. There is no significant change in the trends that coincides with the introduction of the policies. It is likely that they have contributed to the ongoing trend, but this is only speculative.

The heavy emphasis of these policies on caesarean sections (in two out of four countries) has been problematic in a number of ways: caesareans can save lives, but even if utilisation increases it is not easy to know if the medically indicated women have received care. Furthermore, caesareans do not address main causes of maternal deaths as post-partum hemorrhage, complications of abortion, severe malaria, etc. [14]. Moreover, the use of caesareans is heavily skewed to the rich and to urban areas, meaning that the benefits of the funding will almost automatically be biased in favour of the rich. It is an intervention which in some contexts needs boosting, but in other contexts (or for some groups) needs controlling. It can be induced by suppliers and patients for the wrong reasons, and carries medical risks. While policies to reduce the costs of caesareans (which are high cost, potentially catastrophic events from a households perspective) can provide real financial benefits to households, as these have done, from a public health perspective a wider policy covering a range of life-threatening obstetric complications and also the pathway to them (facility deliveries) is preferable. Morocco and Burkina Faso illustrate this approach.

One of the underlying objectives was to reduce the burden of this essential service on households, and thereby change delivery care seeking behaviour. The overall evidence suggests a significant reduction in household payments for the targeted services, ranging from 53 % for caesareans in Benin to 92 % for caesareans in Morocco. The financial protection objective therefore seems to have been met effectively. This is consistent with recent extended cost-effectiveness modelling in countries like Ethiopia [15]. However, even in relation to the package of care which was supposed to be covered, households continued to pay sums which amounted to a small proportion of their overall expenditure in Morocco, intermediate in Burkina and Benin and the large majority in Mali. A significant proportion (0–35 %, depending on site) was unable to pay. Moreover, women reported a lack of certainty about what they should pay or not which not only increases financial problems but also clouds their relationship with providers. This indicates that there is plenty of scope to increase the financial protection offered by the policies.

Furthermore, we found that while financial barriers are significant and are connected to many other barriers (physical, cultural etc.), on their own their reduction does not change behaviour, unless it is connected with a positive shift in other aspects, such as perception of quality and responsiveness. Policies on financial access therefore need to be designed with improvements to these other facets in mind, as an integral part of their design. Behaviour change also has to be measured over a longer period, as habits in relation to significant services such as delivery change slowly.

Looking at a simple comparison of the funds spent by government on the policies versus the estimated gains made by households gives another insight into the value-for-money question. In all three countries for which we have unit cost data (this is missing in Morocco), the average expenditure per delivery was lower than the average gain per household with a delivery. There is therefore a net gain, which probably reflects the payment system and the fact that facilities are providing care without fully recovering their costs. If they are able to do this and still provide adequate care without passing additional costs to women, then the policy is leveraging an efficiency gain in the health system.

Comparing these country experiences with those documented in other countries with similar exemption policies [16–20], we can highlight some strengths. For example:

- The policies have been relatively thoroughly implemented: despite some gaps and lapses, the policies have been put into effect in a serious way
- They have not been affected by budget shortfalls, which undermined the effectiveness of similar policies in countries like Ghana [21]
- They have, in some cases, like Morocco’s, been accompanied by the additional supply-side improvements which are required to meet the additional demands
- There is an underlying support for them, and not only from beneficiaries: most actors within the health system (health district managers, hospital management teams, specialists, nurses and midwives) reacted positively to the policy in interviews. The policy was generally considered to be relevant and important
- They have achieved substantial reductions in household payments, which will over time contribute to poverty reduction and reduced inequalities of access

However, there are also some weaknesses. These include:

- A package of care which in some cases (Mali and to a lesser extent Benin given its utilisation rates) will not address all of the main causes of maternal and early neonatal morbidity and mortality, and whose impact on these can therefore only be expected to be modest, even if well implemented
- Poorly calibrated provider payments for those using fixed payments, which either over-incentivise (in the case of Benin) or under-fund (in the case of Mali). Both of these result in perverse effects and stem in part from poorly understood cost structures
- Lack of clear and well disseminated operating documents, which enable staff and clients to be clear about how the policy will work and what is covered by it
- Too limited attention to the quality of care offered by the facilities covered by the policy; for newborns in particular it has been found to be sub-standard
- Lack of involvement in most cases of managers, staff and communities in developing and monitoring the policy in order to increase ownership and control abuse
- No policy has completely reduced the officially exempted costs to zero; although magnitudes of unwarranted payments vary in scale, all countries need to more effectively regulate providers and stop illicit payments from patients
- By their very design, the policies are unable to address some of the main barriers faced by women, such as the inability to physically access health care. Additional actions are needed to ensure that benefits can be equitable

Wider impacts, positive and negative, intended and unintended, are also to be taken into account in coming to an overall judgement about these policies. We have found a range of these but they vary by context and suggest a variety of outcomes can be expected from these policies, depending on their features but also the context and the institutional and organisational frameworks into which they are introduced. This complexity means that no one simple answer to the overall evaluation question can be produced for all settings.

A range of equity findings have emerged – some positive, like the closing of gaps in coverage – but documenting a continuing set of barriers which discriminate against poor and vulnerable women. These include lack of awareness, lack of real access to facilities, socio-cultural barriers, continuing residual financial barriers, negative staff attitudes and poor implementation of policies, which mean, for example, that elements which are meant to protect the poorest, like the full exemption for the poorest in Burkina Faso, are not funded and put into practice. All of these need to be addressed to ensure that the benefits of these universal policies are distributed according to need.

All four countries show continued large unmet needs for emergency obstetric care for almost all groups (with the exception of better off households in Morocco). For example, the richest have been around 5–6 times more likely to have a caesarean section than the poorest for the whole period studied in Mali. However, even among the richest in the most recent household survey, the caesarean section rate is only 6 %, which is only just entering acceptable levels. Among the poorest there’s a huge unmet need for EMOC.

In addition to the weaknesses of the policies themselves, there are underlying systemic weaknesses that undermine policy effectiveness and fairness, such as lack of effective stewardship at the local level, inadequate communication over the policy with staff and communities, staffing which favours urban and well-connected areas, drugs supply and distribution systems that are not reliable, and poor provider-patient relationships in some areas. Improving these will be key to making these and similar policies operate effectively and in a pro-poor manner.
